# Supplementary material for: Lzts1 controls both neuronal delamination and outer radial glial-like cell generation during mammalian cerebral development
Source: Nat Commun. 2019 Jun 25;10:2780. doi: 10.1038/s41467-019-10730-y (PMC6592889; doi:10.1038/s41467-019-10730-y)
Supplement: Supplementary file 1 — Supplementary Information [file 41467_2019_10730_MOESM1_ESM.pdf]

SUPPLEMENTARY INFORMATION

Lzts1 controls both neuronal delamination and outer radial glial-like cell generation during mammalian cerebral development

Kawaue *et al.*

Supplementary Table 1

Primers for plasmid DNA construction

kzk-Lzts1-F1	5’-CCACCATGGGCAGTGTGAGCAGCCTTATC-3’
Lzts1-B1	5’ -TCAGATCTCAGTGGCTATGATGTCC-3’
kzk-Lzts1-mut-F1	5’ -CCACCATGGGCAGTGTGAGCAGTCTCATATCTGGCCACAG-3’
KpnI-B3-Lzts1	5’ -ACGGTACCGTGATCTCAGTGGCTATGATGTC-3’
kzk-F4-G2A	5’ -ACGAATTCCCACCATGGCCAGTGTGAGCAGCAG-3’
Lzts1-B765	5’ -ACGTCGACCCATCAGAGAACGACAGAGC-3’
FuroLz-F1	5’ -GAATTCTGCAGTCGACCACCATGGGTAGTGTCAGTAGCCTC-3’
FuroLz-R1	5’ -CATTTCTAGCTGATTGGAGAAGGGCATAAGCTTGG-3’
FuroLz-F2	5’ -GGCTCAGAGAAGGGTGCAGTGAGG-3’
FuroLz-R2	5’ -TGACTTCTGGCACACCTCCCACTGGGTCTCTTCCAG-3’
FuroLz-F3	5’ -GTGTGCCAGAAGTCAGGGGAGATC-3’
FuroLz-R3	5’ -CCGCGGTACCGTCGAGATCTCAGTGGCTATGATGTC-3’
kzk-Scrt1-F	5’ -CCACCATGCCCAGGTCCTTCCTGGTTAAGAAGG-3’
Scrt1-R	5’ -CCTAGGCTTGAACCGGGCTGAGCTGC-3’

Supplementary Table 2

Primers for gRNA vectors

Mouse, Lzts1
musLz-gRNA-F608
5'-TTATATATCTTGTGGAAAGGACGAAACACCgGCTGAACCCCCAAAACGGC-3'
musLz-gRNA-R608
5'-ATTTTAACTTGCTATTTCTAGCTCTAAAACGCCGTTTTGGGGGTTCAGCC-3'

Ferret, Lzts1
KO #1
FuroLz-gRNA-F273
5'-TTATATATCTTGTGGAAAGGACGAAACACCGCGGGCTTCACGATACAAGT-3'
FuroLz-gRNA-R273
5'-ATTTTAACTTGCTATTTCTAGCTCTAAAACACTTGTATCGTGAAGCCCGC-3'

FuroLz-gRNA-F477
5'-TTATATATCTTGTGGAAAGGACGAAACACCGAGTGGGGACATAGGCGGCC-3'
FuroLz-gRNA-R477
5'-ATTTTAACTTGCTATTTCTAGCTCTAAAACGGCCGCCTATGTCCCCACTC-3'

FuroLz-gRNA-F1527
5'-TTATATATCTTGTGGAAAGGACGAAACACCGGAGAGTTCTCTGCGTACCA-3'
FuroLz-gRNA-R1527
5'-ATTTTAACTTGCTATTTCTAGCTCTAAAACTGGTACGCAGAGAACTCTCC-3'

KO #2
FuroLz-gRNA-F823
5'-TTATATATCTTGTGGAAAGGACGAAACACCGAGCCTCCAAAACGGCTGGT-3'
FuroLz-gRNA-R823
5'-ATTTTAACTTGCTATTTCTAGCTCTAAAACACCAGCCGTTTTGGAGGCTC-3'

FuroLz-gRNA-F1302
5'-TTATATATCTTGTGGAAAGGACGAAACACCGAAGCTCAGGTCCTACGAGA-3'
FuroLz-gRNA-R1302
5'-ATTTTAACTTGCTATTTCTAGCTCTAAAACTCTCGTAGGACCTGAGCTTC-3'

FuroLz-gRNA-F316
5'-TTATATATCTTGTGGAAAGGACGAAACACCGAGCTCAATCGGTATTCAGA-3'
FuroLz-gRNA-R316
5'-ATTTTA ACTTGCTATTTCTAGCTCTAAAACTCTGAATACCGATTGAGCTC-3'

Ferret, negative control (NC; intron of *GDF5*)
FuroNC-gRNA-F
5'-TTATATATCTTGTGGAAAGGACGAAACACCGTTTTATCGTCAACCCGGGA-3'
FuroNC-gRNA-R
5'-ATTTTAACTTGCTATTTCTAGCTCTAAAACTCCCGGGTTGACGATAAAAC-3'

Supplementary Table 3

Concentration of siRNA and plasmid DNAs

Experiment	siRNA or plasmid DNA	Concentration
**Fig. 2a**		
Neurog1/2	pCAG::Neurog1	0.7 μg μl^{-1}
	pEF::Neurog2	0.7 μg μl^{-1}
	pCAG::EGFP	0.5 μg μl^{-1}
**Fig. 2b, c, Supplementary Fig. 4**		
siRNA#1 or #2	siRNA#1 or #2	100 μM
	pCAG::Neurog1	0.7 μg μl^{-1}
	pEF::Neurog2	0.7 μg μl^{-1}
	pCAG::EGFP3NLS	0.3 μg μl^{-1}
siRNA-NC	siRNA-negative control	100 μM
	pCAG::Neurog1	0.7 μg μl^{-1}
	pEF::Neurog2	0.7 μg μl^{-1}
	pCAG::EGFP3NLS	0.3 μg μl^{-1}
Rescue	siRNA#1	100 μM
	pCAG::Neurog1	0.7 μg μl^{-1}
	pEF::Neurog2	0.7 μg μl^{-1}
	pCAG::EGFP3NLS	0.3 μg μl^{-1}
	pCAG::siRNA-resistant-Lzts1	0.1 μg μl^{-1}
Rescue control	siRNA#1	100 μM
	pCAG::Neurog1	0.7 μgμl^{-1}
	pEF::Neurog2	0.7 μg μl^{-1}
	pCAG::EGFP3NLS	0.3 μg μl^{-1}
	pCAG::Lzts1	0.1 μg μl^{-1}
**Fig. 2d**		
Cre+ lyn-EGFP	siRNA (NC or #1)	100 μM
	pCAG::Neurog1	0.7 μg μl^{-1}
	pEF::Neurog2	0.7 μg μl^{-1}
	pEF::Cre	0.001 μg μl^{-1}
	pEF::LPL-Lyn-EGFP	0.5 μg μl^{-1}
**Fig. 3e–g, j**		
KO	pCAX-hCas9	0.5 μg μl^{-1}
	hU6::gRNA-musLzts1	0.5 μg μl^{-1}
	pCAG::EGFP	0.3 μg μl^{-1}
Control	pCAX-hCas9	0.5 μg μl^{-1}
	pCAG::EGFP	0.5 μg μl^{-1}
**Fig. 3a–d, h–j, Supplementary Fig. 4, 9, 10**		
KD, control	pCAG::EGFP3NLS	0.3 μg μl^{-1}
	siRNA (NC or #1)	100 μM
siRNA#1 +siRNAresistantLzts1 (rescue)	pCAG::EGFP3NLS	0.3 μg μl^{-1}
	siRNA #1	100 μM
	pCAG::siRNA-resistant-Lzts1	0.1 μg μl^{-1}
siRNA#1+Lzts1 (rescue control)	pCAG::EGFP3NLS	0.3 μg μl^{-1}
	siRNA #1	100 μM
	pCAG::Lzts1	0.1 μg μl^{-1}
**Fig. 4 (Gadd45g::d4Venus Tg)**		
KO	pCAX-hCas9	0.5 μg μl^{-1}
	hU6::gRNA-musLzts1	0.5 μg μl^{-1}
	pCAG::dsRed	0.3 μg μl^{-1}
Control	pCAX-hCas9	0.5 μg μl^{-1}
	pCAG::dsRed	0.3 μg μl^{-1}

**Fig. 5b, d**		
Lzts1 1.0, control	pEF::LPL-EGFP	0.5 μg μl^{-1}
	pEF::Cre	0.001 μg μl^{-1}
	pEF::LPL-Lyn-EGFP	0.5 μg μl^{-1}
	with or without	
	pCAG::Lzts1	1.0 μg μl^{-1}
**Fig. 5d–m**		
Control	pCAG::EGFP-3NLS	0.5 μg μl^{-1}
Lzts1 1.0, 0.2	pCAG::Lzts1	1.0 or 0.2 μg μl^{-1}
	pCAG::EGFP-3NLS	0.3 μg μl^{-1}
**Fig. 6a–e, Supplementary Movie 1–4**		
Lzts1 1.0, 2.0	pCAG::Lzts1	1.0 or 2.0 μg μl^{-1}
	pEF::LPL-H2B-mRFP	0.5 μg μl^{-1}
	pEF::Cre	0.001 μg μl^{-1}
	pEF::LPL-Lyn-EGFP	0.5 μg μl^{-1}
Control	pEF::LPL-H2B-mRFP	0.5 μg μl^{-1}
	pEF::Cre	0.001 μg μl^{-1}
	pEF::LPL-Lyn-EGFP	0.5 μg μl^{-1}
**Fig. 7a**		
Control, Lzts1 1.0	pCAG::EGFP	0.5 μg μl^{-1}
	pCAG::mCherry-ZO1	0.5 μg μl^{-1}
	with or without	
	pCAG::Lzts1	1.0 μg μl^{-1}
**Fig. 7b, e, f**		
Lzts1 1.0	pCAG::mCherry-ZO1	0.5 μg μl^{-1}
	pCAG::Lzts1	1.0 μg μl^{-1}
**Fig. 7c, d**		
Control, Lzts1 0.7	pCAG::mCherry-ZO1	0.5 μg μl^{-1}
	with or without	
	pCAG::Lzts1	0.7 μg μl^{-1}
**Fig. 8a-e**		
Lzts1(-F) 0.2	pCAG::Lzts1-F (a–c)	0.2 μg μl^{-1}
	or pCAG::Lzts1 (d, e)	
	pBA::LPL-H2B-EGFP	0.5 μg μl^{-1}
	pCAG::Cre	0.05μg μl^{-1}
Control	pBA::LPL-H2B-EGFP	0.5 μg μl^{-1}
	pCAG::Cre	0.05μg μl^{-1}
**Fig. 9e**		
KO	pBA::LPL-H2B-EGFP	0.5 μg μl^{-1}
	pCAG::Cre	0.1μg μl^{-1}
	pCAX-hCas9	0.5 μg μl^{-1}
	hU6::gRNA-musLzts1	0.5 μg μl^{-1}
Control	pBA::LPL-H2B-EGFP	0.5 μg μl^{-1}
	pCAG::Cre	0.1μg μl^{-1}
	pCAX-hCas9	0.5 μg μl^{-1}
**Fig. 9f**		
KO	pCAX-hCas9	0.5 μg μl^{-1}
	hU6::gRNA-musLzts1	0.5 μg μl^{-1}
	pPB-CAG::PACT-mCyRFP1	0.4 μg μl^{-1}
	pPB-CAG::EGFP-ZO1	0.2 μg μl^{-1}
	pCAX-hyPBase	0.2 μg μl^{-1}
Control	pCAX-hCas9	0.5 μg μl^{-1}
	pPB-CAG::PACT-mCyRFP1	0.4 μg μl^{-1}

	pPB-CAG::EGFP-ZO1	0.2 μg μl^{-1}
	pCAX-hyPBase	0.2 μg μl^{-1}
**Fig. 9g–i**		
KD, control	pCAG::EGFP3NLS	0.3 μg μl^{-1}
	siRNA (NC or #1)	100 μM
Rescue	pCAG::EGFP3NLS	0.3 μg μl^{-1}
	siRNA #1	100 μM
	pCAG::siRNA-resistant-Lzts1	0.1 μg μl^{-1}
Rescue-control	pCAG::EGFP3NLS	0.3 μg μl^{-1}
	siRNA #1	100 μM
	pCAG::Lzts1	0.1 μg μl^{-1}
**Fig. 10b–d, Supplementary Fig. 12**		
KO #1	pCAX-hCas9	0.5 μg μl^{-1}
	hU6::gRNA-furoLzts1-273	0.5 μg μl^{-1}
	hU6::gRNA-furoLzts1-477	0.5 μg μl^{-1}
	hU6::gRNA-furoLzts1-1527	0.5 μg μl^{-1}
	pPB-CAG::2EGFP-3NLS	0.2 μg μl^{-1}
	pCAX-hyPBase	0.2 μg μl^{-1}
KO #2	pCAX-hCas9	0.5 μg μl^{-1}
	hU6::gRNA-furoLzts1-823	0.5 μg μl^{-1}
	hU6::gRNA-furoLzts1-1302	0.5 μg μl^{-1}
	hU6::gRNA-furoLzts1-316	0.5 μg μl^{-1}
	pPB-CAG::2EGFP-3NLS	0.2 μg μl^{-1}
	pCAX-hyPBase	0.2 μg μl^{-1}
Control	pCAX-hCas9	0.5 μg μl^{-1}
	hU6::gRNA-furoNC	0.5 μg μl^{-1}
	pPB-CAG::2EGFP-3NLS	0.2 μg μl^{-1}
	pCAX-hyPBase	0.2 μg μl^{-1}
Cas9	pCAX-hCas9	0.5 μg μl^{-1}
	pPB-CAG::2EGFP-3NLS	0.2 μg μl^{-1}
	pCAX-hyPBase	0.2 μg μl^{-1}
**Supplementary Fig. 5c, d**		
	pCAG::Lzts1-F (or ΔC-Lzts1-F)	1.0 μg μl^{-1}
	pCAG::EGFP	0.3 μg μl^{-1}
**Supplementary Fig. 6**		
Lzts1 1.0	pCAG::Lzts1	1.0 μg μl^{-1}
	pCAG::EGFP	0.3 μg μl^{-1}
Scrt1	pCAG::Scrt1	1.0 μg μl^{-1}
	pCAG::EGFP	0.3 μg μl^{-1}
Control	pCAG::EGFP	0.5 μg μl^{-1}
**Supplementary Fig. 7**	pCAG::Lzts1-F	0, 0.2, 1.0, 2.0 μg μl^{-1}
	pCAG::EGFP	0.5 μg μl^{-1}
**Supplementary 11**		
Lzts1 1.0	pCAG::furoLzts1	1.0 μg μl^{-1}
	pCAG::EGFP	0.3 μg μl^{-1}
Control	pCAG::EGFP	0.5 μg μl^{-1}

Supplementary Fig. 1

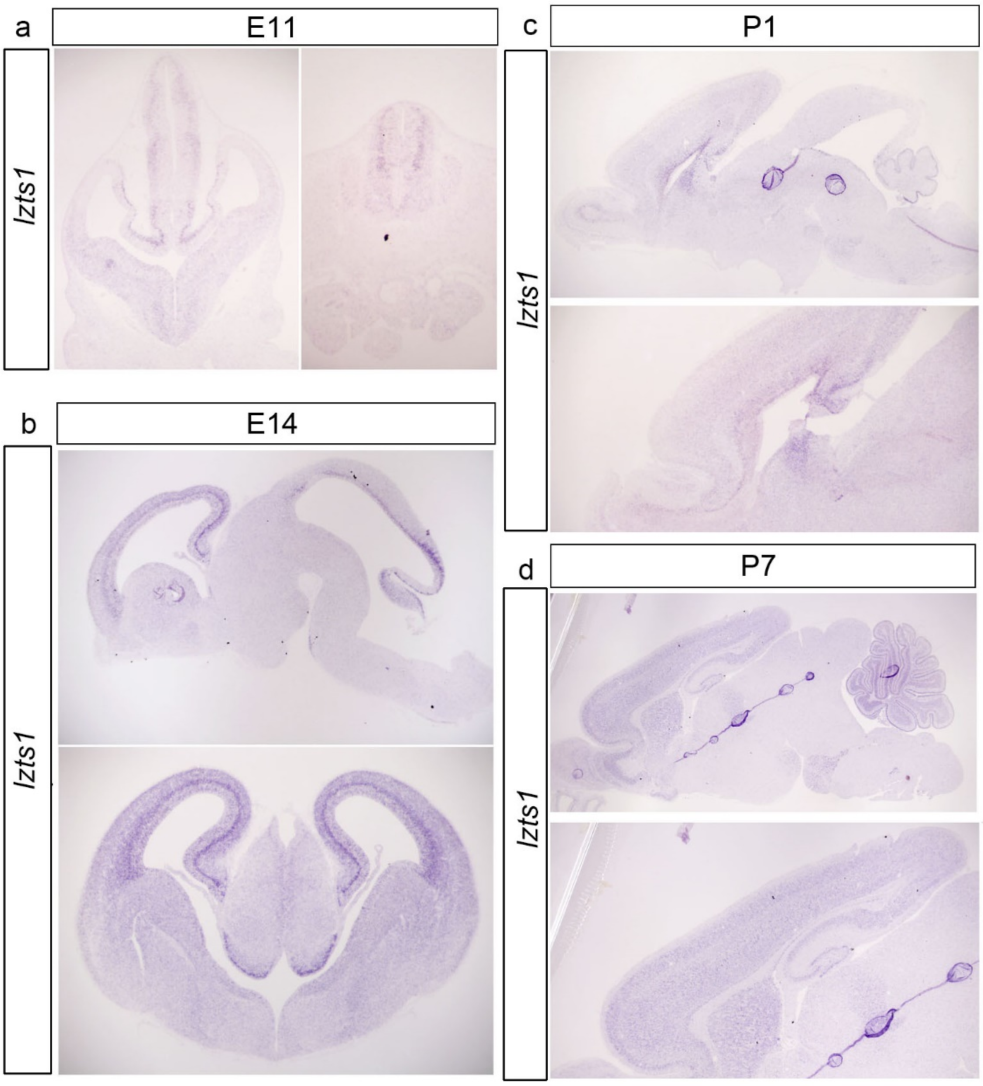

Supplementary Figure 1 (related to Figure 1)

In situ **hybridization of *C230098O21Rik*, an *lzts1* transcript, in the developing mouse CNS.**

E11 (a), E14 (b), P1(c) and P7 (d) mouse brains or spinal cord.

Supplementary Fig. 2

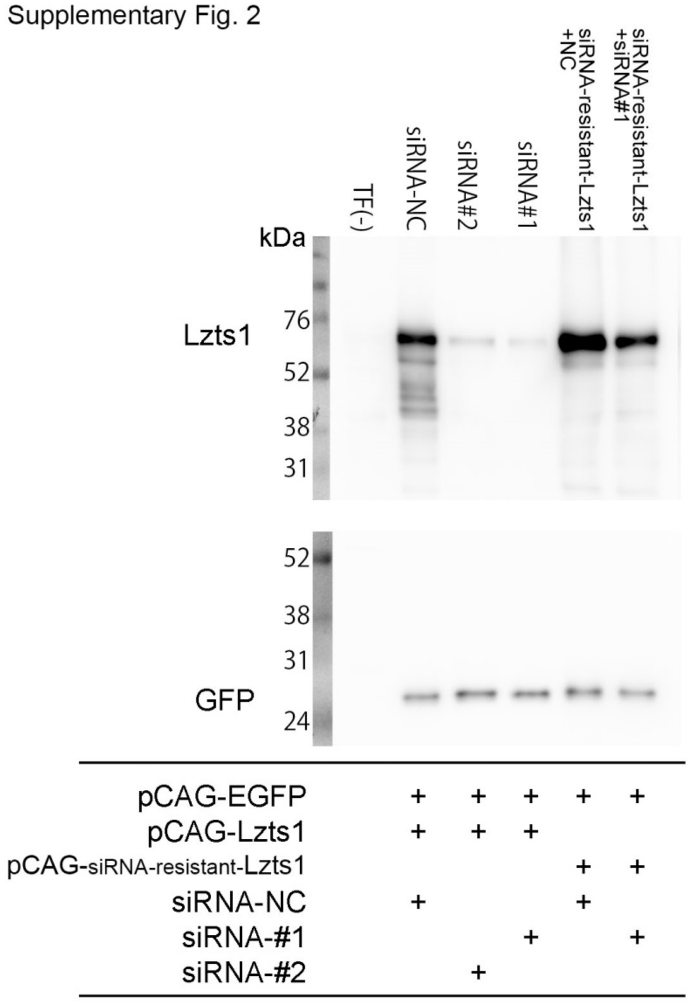

Supplementary Figure 2 (related to Figure 2)

Lzts1-siRNAs repress Lzts1 expression.

The KD efficiency of each siRNA was examined by performing western blots of transfected COS7. siRNA#1 represses Lzts1 expression, but not siRNA#1-resistant-Lzts1 expression. siRNA#2 induces more moderate repression of Lzts1 expression than siRNA#1.

Supplementary Fig. 3

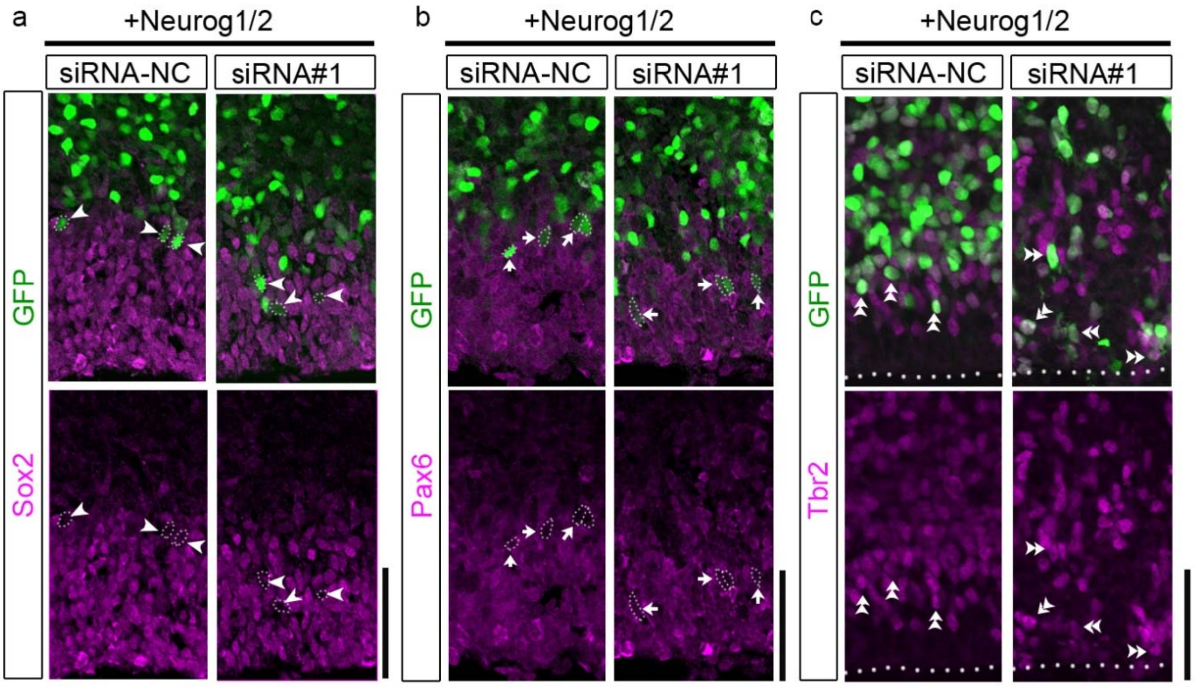

Supplementary Figure 3 (related to Figure 2)

Co-expression of Neurog1 and Neurog2 induces differentiation of electroporated cells.

Neurog1/2 and EGFP with negative control siRNA (siRNA-NC) or Lzts1 siRNA (siRNA#1) were co-electroporated by *in vivo* electroporation at E13, and after 18 h, brains were fixed and stained with anti-Sox2 (a), anti-Pax6 (b), and anti-Tbr2 antibodies (c). Note that most GFP^{+} cells were $Sox2^{-}$ (arrowheads), $Pax6^{-}$ (arrows) and $Tbr2^{+}$ (double-arrowheads) both in the siRNA-NC and siRNA#1 cases. Bar, 50 μm.

Supplementary Fig. 4

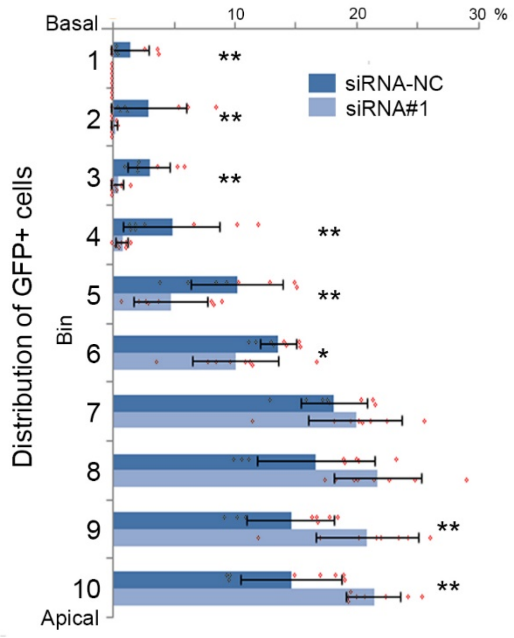

Supplementary Figure 4 (related to Figure 3)

Lzts1 KD retards the radial migration of cells from the apical surface.

In vivo electroporation of the EGFP expression vector with Lzts1-siRNA#1 or siRNA-NC (negative control siRNA) was performed at E13, and the distribution of EGFP$^+$ cells in 10 bins of the cerebral wall was examined at E15 (N=8 sections from 4 embryos were analyzed per experiment, ** p<0.01 and * p<0.05, Brunner-Munzel test). Source data are provided as a Source Data file.

Supplementary Fig. 5

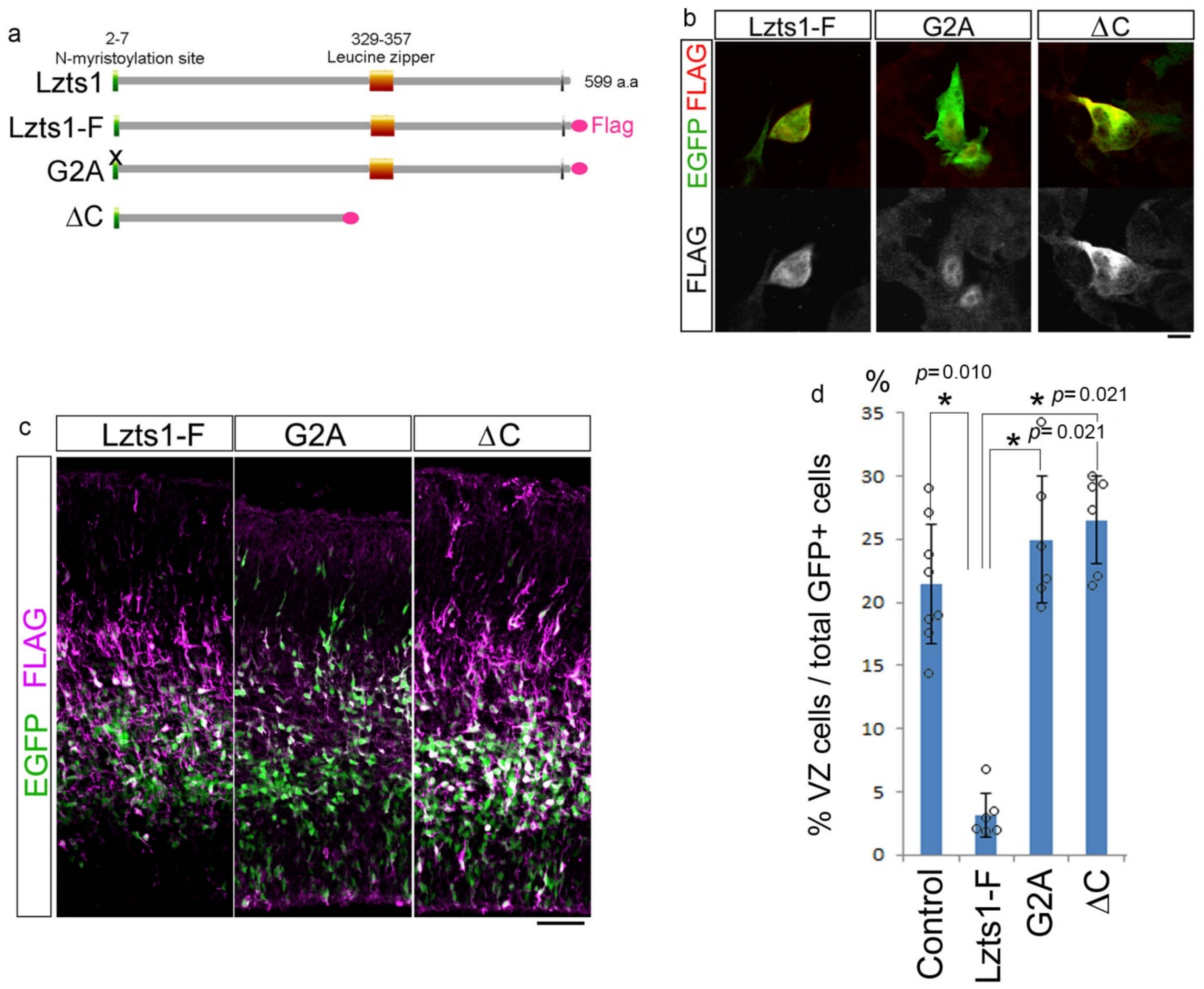

Supplementary Figure 5 (related to Figure 5)

Both the myristoylation site and C-terminal half of Lzts1 are required for positioning cells outside the VZ.

a) Construction of the mutant forms of Lzts1.

b) The G2A mutation in the myristoylation site localized Lzts1 to the nucleus in Neuro2a cells. Bar, 10 μm.

c and **d)** Both the myristoylation site and C-terminal half of Lzts1 are required to reduce the number of VZ cells after forced expression. *In vivo* electroporation was performed at E13 (at a plasmid concentration of 1.0 μg μl^{-1}), and the position of $EGFP^{+}$ cells was examined at E15 (mean ± s.d., N=8, 6, 6, and 6 sections from 4, 3, 3, and 3 embryos, respectively; Steel-Dwass test). Bar, 50 μm. Source data are provided as a Source Data file.

Supplementary Fig. 6

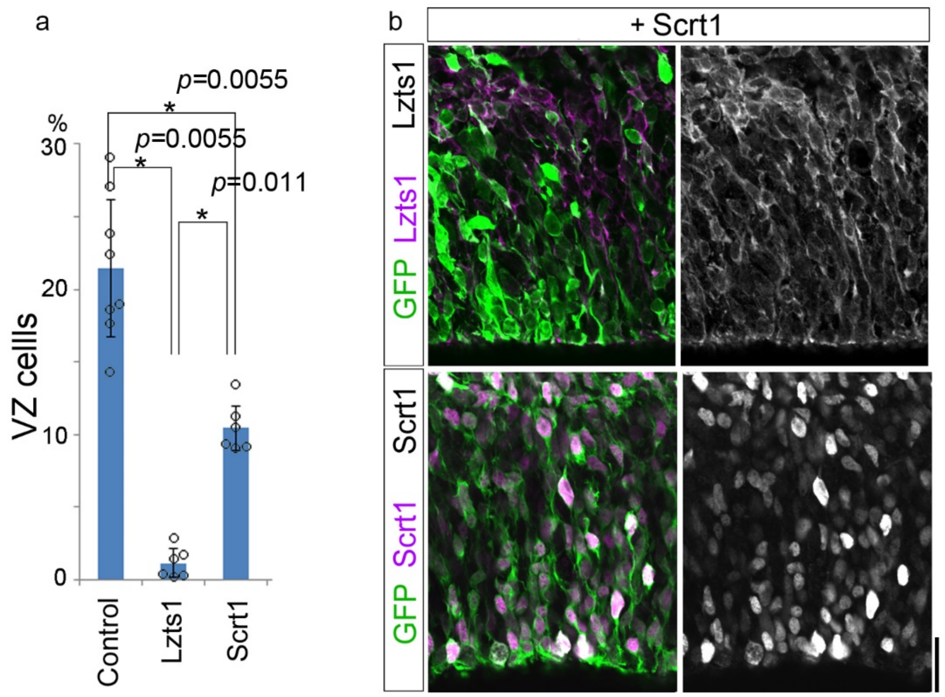

Supplementary Figure 6 (related to Figure 5)

Scrt1 overexpression does not increase Lzts1 expression.

a) Both Lzts1-expressing cells and Scrt1-expressing cells migrate outside the VZ. The percentage of $EGFP^+$ cells present in the VZ was examined 2 days after E13 electroporation; data are presented as means ± s.d., Steel-Dwass test, N=8, 6, and 6 sections from 4, 3, and 3 embryos, respectively.

b) Forced expression of Scrt1 does not increase Lzts1 immunoreactivity. IHC for Scrt1 confirmed that the $EGFP^+$ cells were $Scrt1^+$. The analysis was performed 1 day after the electroporation at E13. Bar, 20 μm. Source data are provided as a Source Data file.

Supplementary Fig. 7

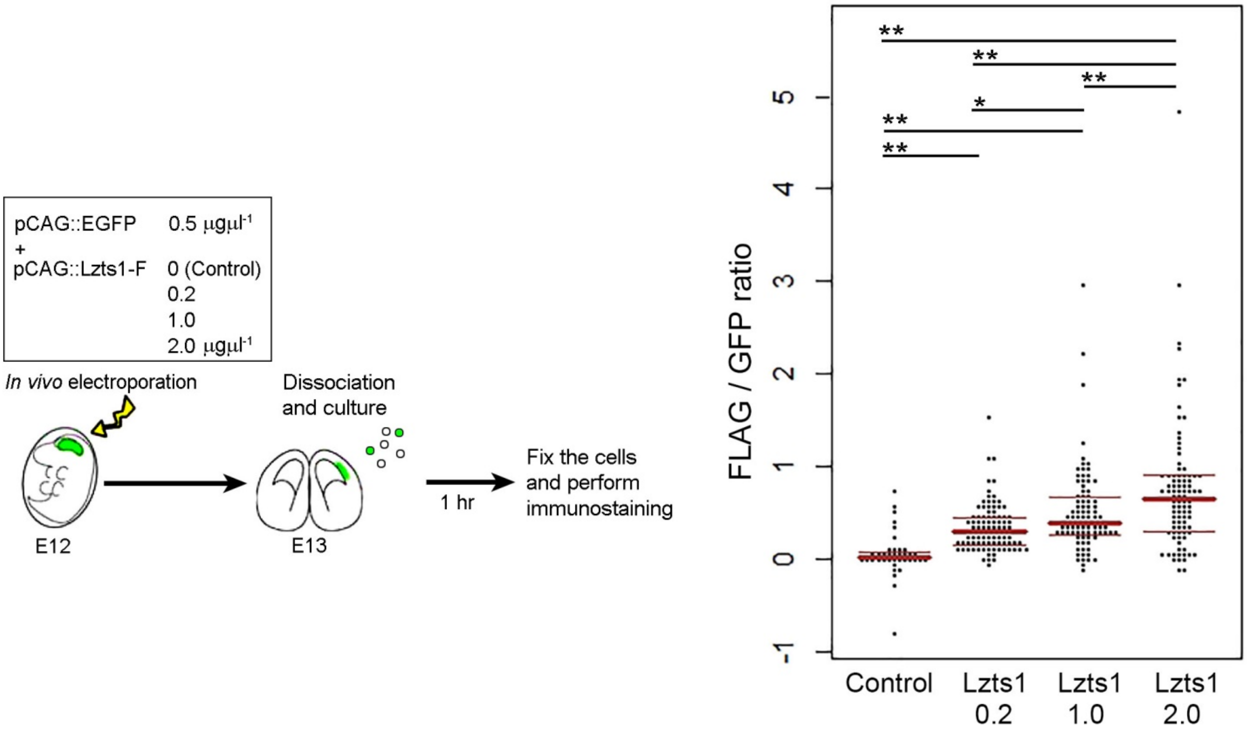

Supplementary Figure 7 (related to Figure 6)

Lzts1 expression levels in the cells were positively correlated with the plasmid vector concentration introduced during the *in vivo* electroporation.

Different concentrations of the Flag-tagged Lzts1-expression vector were co-electroporated with EGFP expression vectors. One day after electroporation, the cerebral walls were dissociated, cultured for 1 hr and stained with anti-Flag and anti-GFP antibodies after fixation. The Flag/GFP ratio was calculated in each GFP^+ cell. * $p<0.05$ and ** $p<0.01$ (Steel-Dwass test, N=38, 98, 88, 92 cells from two embryos and 4 wells per condition; medians with Q1 and Q3 values are shown).

Supplementary Fig. 8

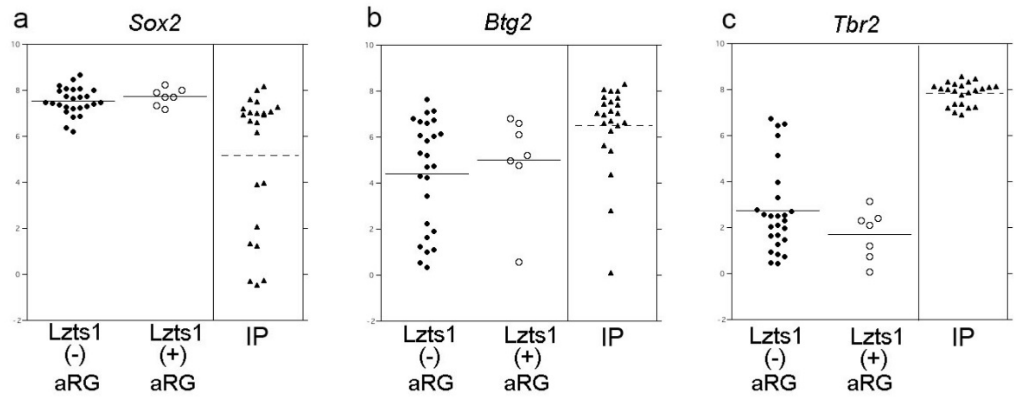

Supplementary Figure 8 (related to Figure 9)

Variations in gene expression in E14 aRGs.

Variations in gene expression in *lzts1*$^+$ aRGs (N=7) and *lzts1*$^-$ aRGs (N=26). No significant differences were detected in *sox2* (*p*=0.45) (**a**), *btg2* (*p*=0.63) (**b**) and *tbr2* (*p*=0.21) (**c**) expression (permuted Brunner-Munzel test, bars indicate means). As the references of the expression levels in the differentiating cells, data from IPs are also indicated. Data from single-cell transcriptome profiles are shown [1].

Supplementary Fig. 9

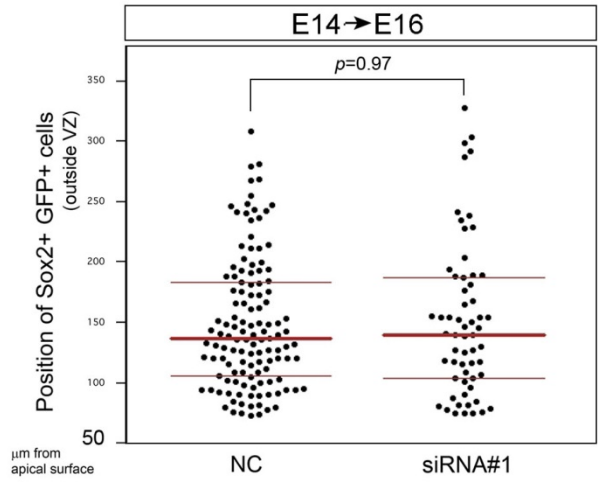

Supplementary Figure 9 (related to Figure 9)

Lzts1 KD does not alter the distribution of SVZ/IMZ Sox2+ cells.

Dot plots indicate the distribution of $Sox2^{+}$ GFP^{+} cells outside the VZ. *In vivo* electroporation of siRNAs with the GFP expression vector was performed in E14 mouse embryos, and the brains were examined after two days. No significant differences in the median values were observed between control and KD tissues (Wilcoxon rank sum test, p=0.97, N=125 and 60 cells from 4 and 4 embryos, respectively, medians with Q1 and Q3 values are shown).

Supplementary Fig. 10

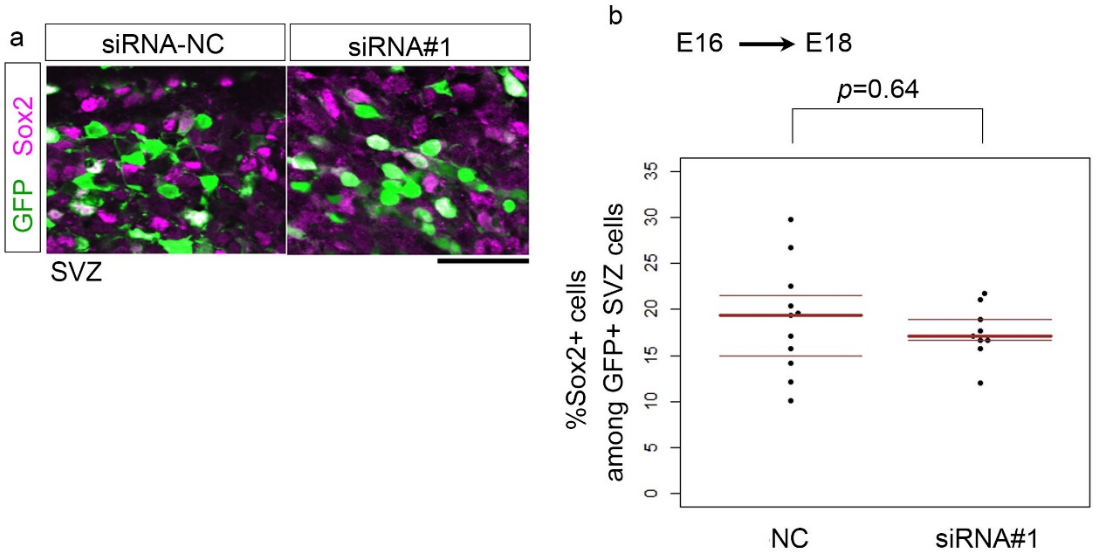

Supplementary Figure 10 (related to Figure 9)

Lzts1 KD did not perturb the generation of $Sox2^+$ SVZ cells from late progenitor cells.

The negative control siRNA (NC) or Lzts1-siRNA (siRNA#1) was electroporated *in vivo* at E16 along with EGFP expression vectors and the brain sections were examined at E18.

a) Some electroporated cells in the SVZ were $Sox2^+$ at E18. Bar, 30 μm.

b) Plot showing the percentage of $Sox2^+$ cells among the GFP+ SVZ cells. No significant difference was detected between the median values for control and KD cells. N=11 and 9 sections from 3 and 4 embryos, respectively, Brunner-Munzel test; medians with Q1 and Q3 values are shown. Source data are provided as a Source Data file.

Supplementary Fig. 11

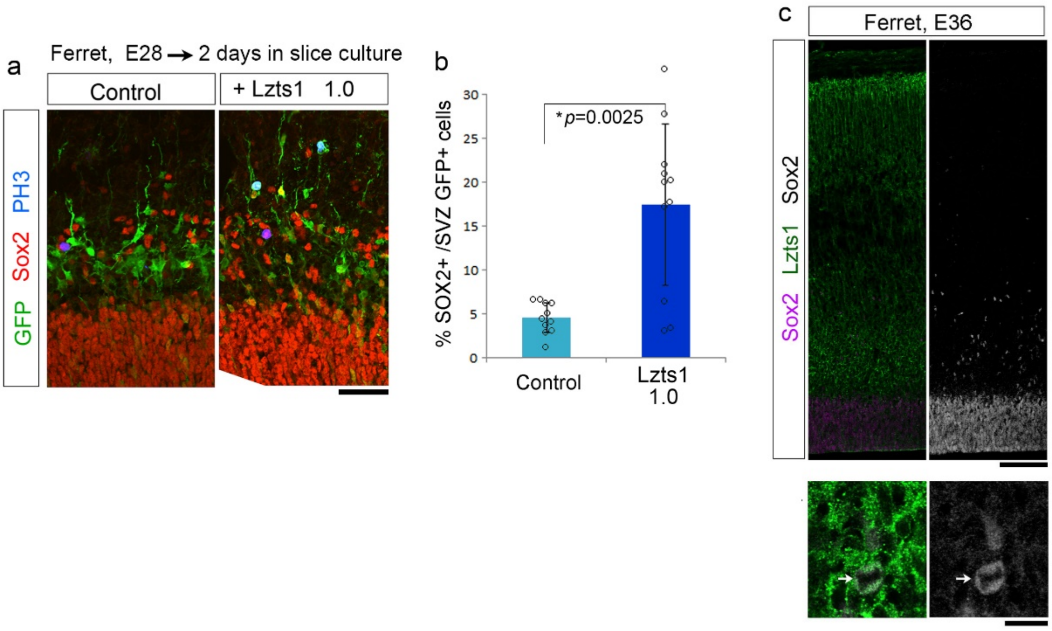

Supplementary Figure 11 (related to Figure 10)

Forced expression of ferret Lzts1 increases the percentage of Sox2⁺ cells in the SVZ.

a) The ferret Lzts1 expression vector was electroporated *ex vivo* at E28. After 48 hrs of slice culture, slices were fixed and stained with anti-GFP, anti-Sox2, and anti-PH3 antibodies. Bar, 50 μm.

b) The percentage of $Sox2^+$ cells among SVZ GFP^+ cells was significantly increased following ferret Lzts1 expression (means ± s.d., N=11 slices from two hemispheres per condition, p=0.0025, Brunner-Munzel test).

c and **c')** IHC for Sox2 and Lzts1 in the E36 ferret cerebral wall (without electroporation). Bar, 100 μm.

c') Magnified view of the SVZ. The arrow indicates an $Lzts1^+$ $Sox2^+$ mitotic cell. Bar, 10 μm. Source data are provided as a Source Data file.

Supplementary Fig. 12

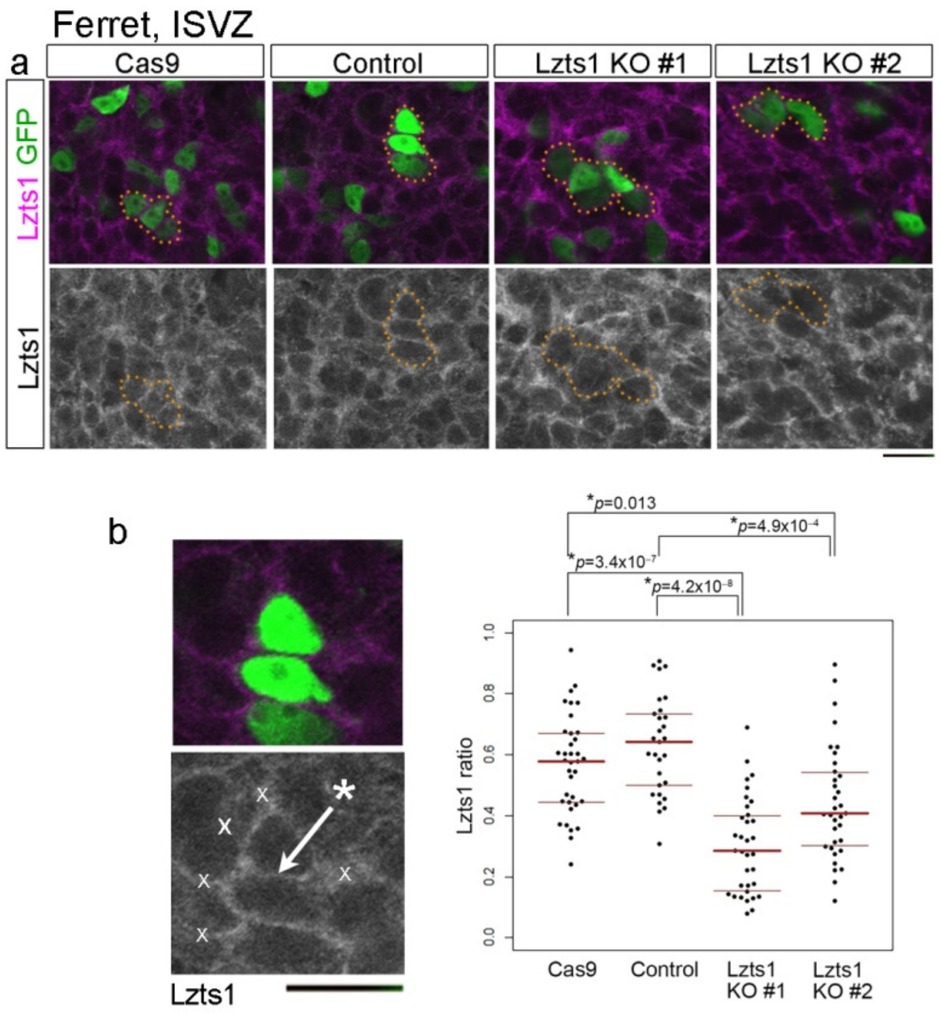

Supplementary Figure 12 (related to Figure 10)

CRISPR/Cas9-mediated KO of Lzts1 in the embryonic ferret brain.

In vivo electroporation of Cas9, gRNA and EGFP-3NLS (EGFP with nuclear localization signals) expression vectors was performed at E32 in ferret embryos, and brains were examined at E38. Four types of experiments were performed, i.e., Cas9 (negative control, without gRNA), control (with the gRNA for a ferret genomic sequence not related to lzts1), 'KO#1' and 'KO#2' (each with mixture of 3 different gRNAs for ferret lzts1).

a) Lzts1 and GFP immunostaining of the ISVZ in the E38 brains.

b) Since Lzts1 is mainly localized to the cytoplasm or cell cortex, we examined the anti-Lzts1 fluorescence signals at the cellular junction (*) where the both neighboring cells were GFP^+ in ISVZ and compared the values to the neighboring cells (x) to examine the Lzts1 KO efficiency. The gRNAs of KO#1 more strongly suppressed Lzts1 expression than those of KO#2. N=34, 29 and 36 cellular junctions from 3 embryos per experiment, Steel-Dwass test, medians with Q1 and Q3 values are shown. Bar, 10 μm.

Supplementary Fig. 13

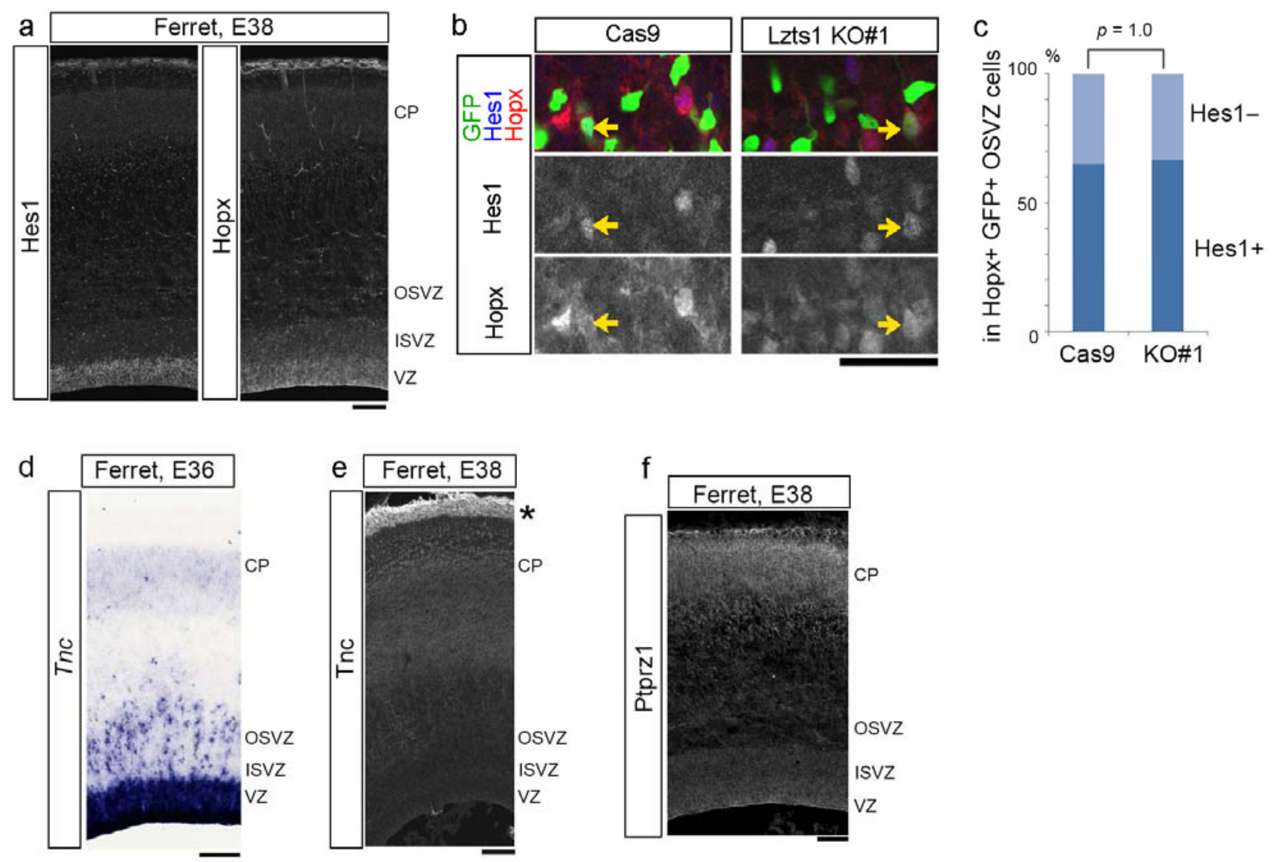

Supplementary Figure 13 (related to Figure 10)

The human oRG-related molecules Hopx, Tnc, and Ptprz1 [2] are not oRG-specific in ferret brains.

a) Anti-Hes1 and anti-Hopx IHC of the ferret E38 cerebral wall. In addition to some OSVZ cells, VZ cells are also immunoreactive for the anti-Hopx antibody, indicating that aRGs also expressed Hopx. Note that weak and broad anti-Hopx immunoreactivity was observed in the ISVZ, which contains many neuronally differentiating cells.

b) Both in the Cas9 and Lzts1 KO#1 cases, all Hes1^{+}GFP^{+} cells in the OSVZ were Hopx^{+} (yellow arrows) (32 and 33 Hes1^{+}GFP^{+} cells examined from 5 hemispheres).

c) Lzts1 KO does not decrease %Hes1^{+}/Hopx^{+}GFP^{+} OSVZ cells, suggesting that Lzts1 KO does not reduce Hes1 expression itself in the individual oRGs; rather, Lzts1 KO reduces the number of newly generated oRGs (40 and 57 Hopx^{+}GFP^{+} OSVZ cells examined, from each 5 hemispheres; two-sided Fisher's exact test) (see also Fig. 10).

d, e) *Tnc* mRNA ISH (d) and anti-Tnc IHC (e) in the ferret brain. Strong immunoreactivity was observed in layer I (asterisk in [e]).

f) Anti-Ptprz1 IHC of the ferret brain. Immunoreactivity of the VZ suggests that the aRGs express Ptprz1. Bars, 100 μm in (a, d-f) and 30 μm in (b).

Supplementary Fig.14

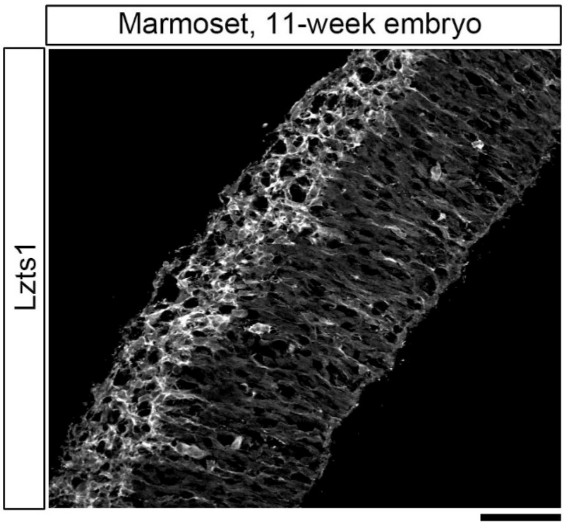

Supplementary Figure 14 (related to Figure 10)

Lzts1 expression in the marmoset brain.

Lzts1 IHC in the cerebral wall of an 11-week marmoset embryo. Bar, 50 μm.

Supplementary Fig. 15

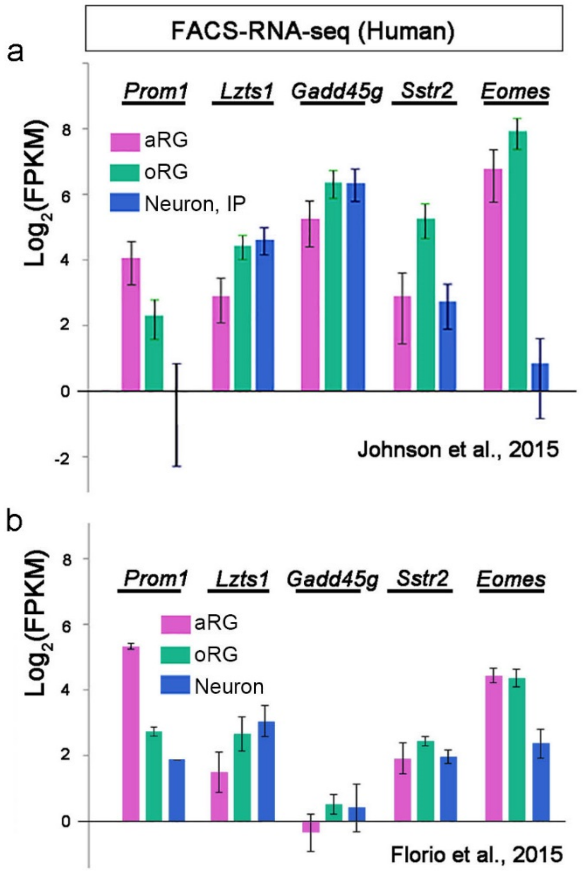

Supplementary Figure 15 (related to Figure 10)

***Lzts1* expression in human oRGs.**

Lzts1 is expressed in human neural progenitor cells in the aRG < oRG pattern.

a) FACS-based RNA-seq data from human neocortical progenitor cells derived from an 18-19 w fetus (aRG: $Glast^+Lex^+Prom1^{high}$, oRG: $Glast^+Lex^+Prom1^{low}$, Neuron+IP: $Glast^-Lex^-Prom1^-$) (GEO database: GSE66217) [3]. The error bar indicates the limit of the 95% FPKM confidence interval.

b) FACS-based RNA-seq data from a wpc 13 w fetus (aRG: DiI^+ $Prom1^+$ S-G2-M-DNA-dye^+, oRG: DiI^+ $Prom1^-$ S-G2-M-DNA-dye^+, Neuron: DiI^- $Prom1^-$ S-G2-M-DNA-dye^-) (GEO database: GSE65000) [4]. Mean ± s.d.

Supplementary Fig. 16

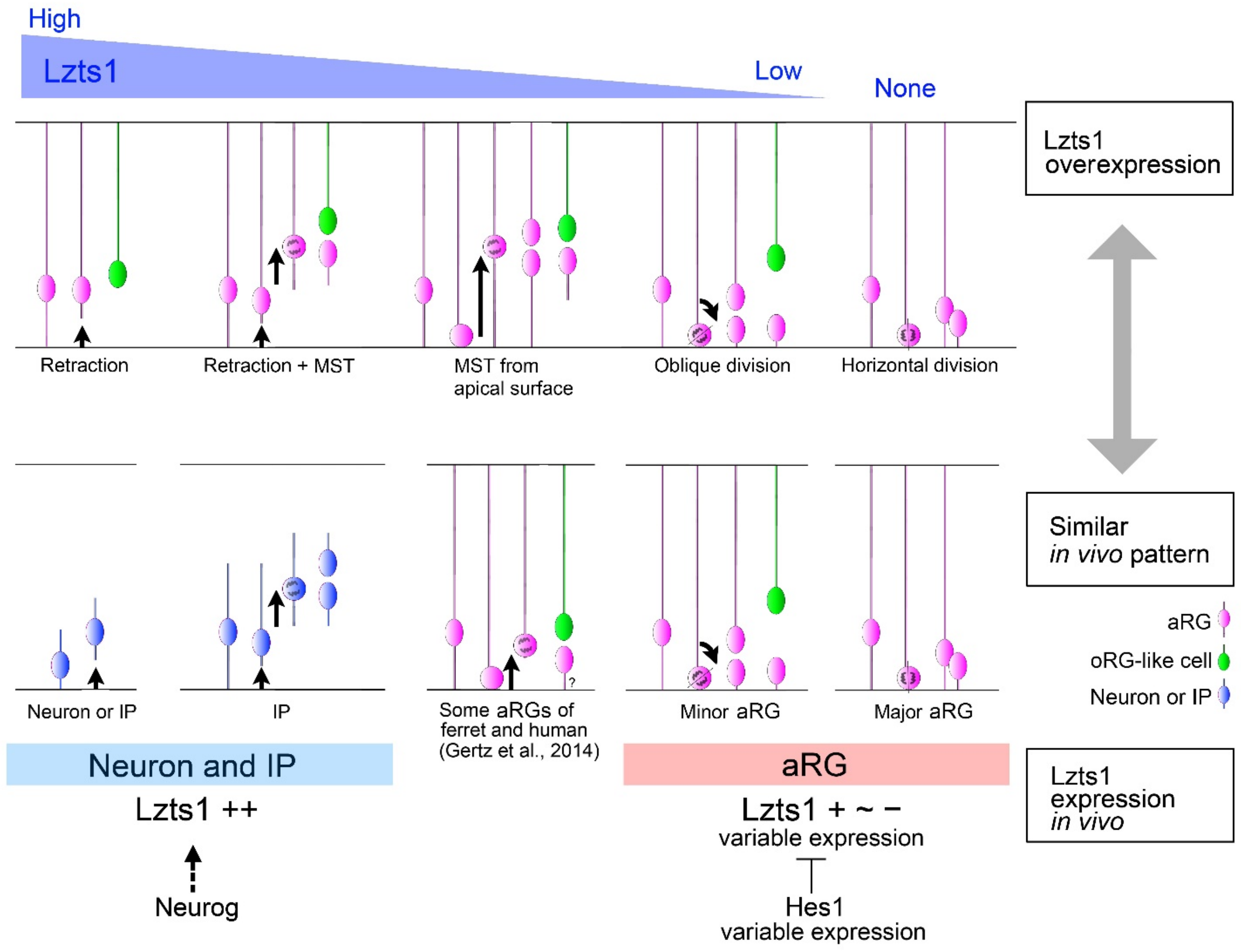

Supplementary Figure 16 (related to Figure 7 and 10)

Lzts1 controls both neuronal delamination and oRG generation as a master modulator of the cytoskeleton.

Lzts1 expression levels correlate with diverse cellular behaviors. Each phenotype observed in overexpressing cells reasonably corresponds to the *in vivo* cellular behaviors mediated by the Lzts1 expression level. Note that 'MST from the apical surface'-like movement has been reported in ferret and human brains [5], but not in mouse brains. *In vivo*, Lzts1 is expressed at high levels in differentiating cells, including nascent neurons and IPs, whereas in the aRG, Lzts1 exhibits variable and weak expression due to the variable Hes1 expression that represses its expression.

aRG, apical radial glial cell (=apical progenitor cell, AP or vRG)

IP, intermediate progenitor cell (=basal progenitor cell, BP)

oRG, outer radial glial cell (=basal RG, bRG)

Supplementary References

1. Kawaguchi, A., *et al.* Single-cell gene profiling defines differential progenitor subclasses in mammalian neurogenesis. *Development* **135**, 3113-3124 (2008).
2. Pollen, A.A., *et al.* Molecular Identity of Human Outer Radial Glia during Cortical Development. *Cell* **163**, 55-67 (2015).
3. Johnson, M.B., *et al.* Single-cell analysis reveals transcriptional heterogeneity of neural progenitors in human cortex. *Nat Neurosci* **18**, 637-646 (2015).
4. Florio, M., *et al.* Human-specific gene ARHGAP11B promotes basal progenitor amplification and neocortex expansion. *Science* **347**, 1465-1470 (2015).
5. Gertz, C.C., Lui, J.H., LaMonica, B.E., Wang, X. & Kriegstein, A.R. Diverse behaviors of outer radial glia in developing ferret and human cortex. *J Neurosci* **34**, 2559-2570 (2014).
