# Supplementary material for: Lzts1 controls both neuronal delamination and outer radial glial-like cell generation during mammalian cerebral development
Source: Nat Commun. 2019 Jun 25;10:2780. doi: 10.1038/s41467-019-10730-y (PMC6592889; doi:10.1038/s41467-019-10730-y)
Supplement: Supplementary file 3 — Description of Additional Supplementary Files [file 41467_2019_10730_MOESM3_ESM.pdf]

Description of Additional Supplementary Files

File Name: Supplementary Movie 1
Description: Live imaging of an *lzts1*-overexpressing aRG-like cell showing the retraction of the apical process (Type A). (.mov file, related to Figure 6)

File Name: Supplementary Movie 2
Description: Live imaging of an *lzts1*-overexpressing aRG-like cell showing the retraction of the apical process followed by MST (Type B). (.mov file, related to Figure 6)

File Name: Supplementary Movie 3
Description: Live imaging of an *lzts1*-overexpressing aRG-like cell showing the 'MST from the apical surface' behavior after apical INM during G2 phase (Type C). (.mov file, related to Figure 6)

File Name: Supplementary Movie 4
Description: Live imaging of an aRG-like cell that underwent normal INM and divided at the apical surface (Normal). (.mov file, related to Figure 6)
