# Supplementary material for: Lzts1 controls both neuronal delamination and outer radial glial-like cell generation during mammalian cerebral development
Source: Nat Commun. 2019 Jun 25;10:2780. doi: 10.1038/s41467-019-10730-y (PMC6592889; doi:10.1038/s41467-019-10730-y)
Supplement: Supplementary file 8 — Reporting Summary [file 41467_2019_10730_MOESM8_ESM.pdf]

nature research

Corresponding author(s): Ayano Kawaguchi

Last updated by author(s): May 9, 2019

Reporting Summary

Nature Research wishes to improve the reproducibility of the work that we publish. This form provides structure for consistency and transparency in reporting. For further information on Nature Research policies, see Authors & Referees and the Editorial Policy Checklist.

Statistics

For all statistical analyses, confirm that the following items are present in the figure legend, table legend, main text, or Methods section.

n/a	Confirmed	
☐	☒	The exact sample size (*n*) for each experimental group/condition, given as a discrete number and unit of measurement
☐	☒	A statement on whether measurements were taken from distinct samples or whether the same sample was measured repeatedly
☐	☒	The statistical test(s) used AND whether they are one- or two-sided<br>*Only common tests should be described solely by name; describe more complex techniques in the Methods section.*
☐	☒	A description of all covariates tested
☐	☒	A description of any assumptions or corrections, such as tests of normality and adjustment for multiple comparisons
☐	☒	A full description of the statistical parameters including central tendency (e.g. means) or other basic estimates (e.g. regression coefficient) AND variation (e.g. standard deviation) or associated estimates of uncertainty (e.g. confidence intervals)
☒	☐	For null hypothesis testing, the test statistic (e.g. *F*, *t*, *r*) with confidence intervals, effect sizes, degrees of freedom and *P* value noted<br>*Give P values as exact values whenever suitable.*
☒	☐	For Bayesian analysis, information on the choice of priors and Markov chain Monte Carlo settings
☒	☐	For hierarchical and complex designs, identification of the appropriate level for tests and full reporting of outcomes
☒	☐	Estimates of effect sizes (e.g. Cohen's *d*, Pearson's *r*), indicating how they were calculated

Our web collection on statistics for biologists contains articles on many of the points above.

Software and code

Policy information about availability of computer code

Data collection	Spindle orientation was measured by the R package as previously reported (Delaunay et al., 2015). This "utilFuncs.R" script has been provided as text format at https://doi.org/10.3389/fncel.2015.00033.
Data analysis	We used open software 'R' x64 3.5.1. to analyze the data.

For manuscripts utilizing custom algorithms or software that are central to the research but not yet described in published literature, software must be made available to editors/reviewers. We strongly encourage code deposition in a community repository (e.g. GitHub). See the Nature Research guidelines for submitting code & software for further information.

Data

Policy information about availability of data

All manuscripts must include a data availability statement. This statement should provide the following information, where applicable:

- Accession codes, unique identifiers, or web links for publicly available datasets
- A list of figures that have associated raw data
- A description of any restrictions on data availability

The source data underlying Figs. 2-6, 9, 10 and Supplementary Figs. 4-6, 10 and 11 are provided as a Source Data file. Other datasets generated during the current study are available from the corresponding authors on reasonable request.

Field-specific reporting

Please select the one below that is the best fit for your research. If you are not sure, read the appropriate sections before making your selection.

☒ Life sciences ☐ Behavioural & social sciences ☐ Ecological, evolutionary & environmental sciences

For a reference copy of the document with all sections, see nature.com/documents/nr-reporting-summary-flat.pdf

Life sciences study design

All studies must disclose on these points even when the disclosure is negative.

Sample size	No statistical methods were used to predetermine the sample size due to experimental limitations, but the sample sizes used here were similar to those described in related previous studies.
Data exclusions	No data were excluded from the analyses.
Replication	All attempts at replication were successful.
Randomization	No randomization was used because of the experimental limitations; but in the case of prenatal experiments by in vivo electroporation, we took in the control and experimental samples in the same litters.
Blinding	Blindings were performed for capture images and quantification of Sox2+GFP+ cells (Fig 9) and Hes1+GFP+ cells (Fig 10) in the SVZ.

Reporting for specific materials, systems and methods

We require information from authors about some types of materials, experimental systems and methods used in many studies. Here, indicate whether each material, system or method listed is relevant to your study. If you are not sure if a list item applies to your research, read the appropriate section before selecting a response.

Materials & experimental systems

n/a	Involved in the study	
☐	☒	Antibodies
☐	☒	Eukaryotic cell lines
☒	☐	Palaeontology
☐	☒	Animals and other organisms
☒	☐	Human research participants
☒	☐	Clinical data

Methods

n/a	Involved in the study	
☒	☐	ChIP-seq
☒	☐	Flow cytometry
☒	☐	MRI-based neuroimaging

Antibodies

Antibodies used	We used the first antibodies as follows: rabbit anti-Lzts1 pAb (HPA006294, Sigma-Aldrich, 1:800 [Fig. 1c]) (20878-1-AP, Proteintech Group, Rosemont, USA; recommended), mouse anti-ZO1 mAb (33-9100, Life Technologies), mouse anti-BrdU mAb (B2531, Sigma-Aldrich), rat anti-BrdU mAb (NB500-169, Novus Biologicals), chicken anti-GFP pAb (GFP-1020, Aves Labs, Tigard, USA), rabbit anti-GFP pAb (598, MBL, Nagoya, Japan), rat anti-GFP mAb (GF090R, Nacalai Tesque, Kyoto, Japan), rabbit anti-Tbr2 pAb (ab23345, Abcam, Cambridge, UK), rabbit anti-Tbr2 mAb (ab183991, Abcam), rabbit anti-Sox2 pAb (ab97959, Abcam), mouse eFluor 660-conjugated anti-Sox2 mAb (50-9811-82, Thermo Fisher Scientific), rabbit anti-Pax6 pAb (PRB-278P, Covance, Princeton, USA), rabbit anti-Pax6 mAb (ab195045, Abcam), rat anti-PH3 mAb (HTA28, ab10543, Abcam), rabbit anti-PH3 pAb (06-570, Sigma-Aldrich), mouse anti-Ki67 mAb (NCL-L-Ki67-MM1, Leica Biosystems, Wetzlar, Germany), rabbit anti-RFP pAb (PM005, MBL), rat anti-RFP mAb (5F8, Chromo Tek, Planegg, Germany), rabbit anti-Scrt1 pAb (HPA045265, Sigma-Aldrich), rabbit anti-N-cadherin pAb (M142, TAKARA Bio Inc, Kusatsu, Japan), mouse anti-phospho-myosin light chain 2 mAb (S19, Cell Signaling Technology, Danvers, USA.), rabbit anti-gamma-tubulin pAb (T5192, Sigma-Aldrich), goat anti-gamma-tubulin pAb (sc-7396, Santa Cruz Biotechnology), mouse anti-alpha-tubulin mAb (clone DM1A, T6199, Sigma-Aldrich), mouse anti-beta-actin mAb (clone AC-74, A2228, Sigma-Aldrich), rat anti-Hes1 mAb (NM1, MBL), rabbit anti-Flag pAb (DYKDDDK Tag antibody, PA1-984B, Thermo Fisher Scientific), rabbit anti-HOPX pAb (FL-73, sc-30216, Santa Cruz Biotechnology), rabbit anti-Tenascin C mAb (ab108930, Abcam), and mouse anti-PTP zeata mAb (ab126497, Abcam).<br>Rabbit anti-LGN pAb was previously described (Konno et al., 2008).
Validation	The data for validation of the commercially available antibodies were provided at the manufactures' web sites. Validation of the anti-LGN pAb was described in the previous paper (Konno et al., 2008).

Eukaryotic cell lines

Policy information about cell lines

Cell line source(s)	RIKEN, BRC (cell bank)
Authentication	The cell lines used here were not authenticated.
Mycoplasma contamination	The cell lines used here were not tested for Mycoplasma contamination.
Commonly misidentified lines (See ICLAC register)	None

Animals and other organisms

Policy information about studies involving animals; ARRIVE guidelines recommended for reporting animal research

Laboratory animals	CD1 mice (Crlj:ICR and Slc:ICR) were used throughout the mouse experiments. Tbr2::EGFP (Eomes::EGFP) BAC transgenic mice (Fig. 1e) were generated by the GENSAT Project, NINDS Contract #N01NS02331 to Rockefeller University (New York, USA). The generation and characterization of Gadd45g::d4Venus transgenic mice has been reported. R26-ZO1-EGFP mice (Accession No. CDB0260K, http://www2.clst.riken.jp/arg/reporter_mice.html) were used to visualize the localization of ZO1. Ferrets were purchased from Marshall Bioresources (New York, USA). To time animal pregnancies, we defined the date when a vaginal plug was observed as embryonic day (E) 0. The sex of the embryos used was not examined.
Wild animals	This study did not involve wild animals.
Field-collected samples	This study did not include field-collected samples.
Ethics oversight	All animal experiments were performed in accordance with institutional guidelines (Nagoya University and RIKEN BDR).

Note that full information on the approval of the study protocol must also be provided in the manuscript.
